# The mTOR Pathway: A Common Link Between Alzheimer’s Disease and Down Syndrome

**DOI:** 10.3390/jcm13206183

**Published:** 2024-10-17

**Authors:** Abigail J. Wohlfert, Jeremiah Phares, Ann-Charlotte Granholm

**Affiliations:** 1 Department of Modern Human Anatomy and Cell & Developmental Biology, University of Colorado Anschutz Medical Campus, Aurora, CO 80045, USA; abigail.wohlfert@cuanschutz.edu; 2 Department of Neurosurgery, University of Colorado Anschutz Medical Center, Aurora, CO 80045, USA; jeremiah.phares@cuanschutz.edu

**Keywords:** mTOR, intellectual disability, neurodegeneration, spatial transcriptomics, proteomics, metabolomics

## Abstract

Down syndrome (DS) is a chromosomal condition that causes many systemic dysregulations, leading to several possible age-related diseases including Alzheimer’s disease (AD). This may be due to the triplication of the Amyloid precursor protein (APP) gene or other alterations in mechanistic pathways, such as the mTOR pathway. Impairments to upstream regulators of mTOR, such as insulin, PI3K/AKT, AMPK, and amino acid signaling, have been linked to amyloid beta plaques (Aβ) and neurofibrillary tangles (NFT), the most common AD pathologies. However, the mechanisms involved in the progression of pathology in human DS-related AD (DS-AD) are not fully investigated to date. Recent advancements in omics platforms are uncovering new insights into neurodegeneration. Genomics, spatial transcriptomics, proteomics, and metabolomics are novel methodologies that provide more data in greater detail than ever before; however, these methods have not been used to analyze the mTOR pathways in connection to DS-AD. Using these new techniques can unveil unexpected insights into pathological cellular mechanisms through an unbiased approach.

## 1. Introduction

Down syndrome (DS) is a chromosomal alteration caused by a complete or partial triplication of chromosome 21. This condition is characterized by intellectual disability, cardiovascular issues, and systemic dysregulation [1]. DS is also characterized by an elevated risk for Alzheimer’s Disease (AD), the most prevalent neurodegenerative disease [2]. AD affects numerous regions of the brain, leading to deficits in judgment, memory loss, and emotional and behavioral dysregulation. Autosomal dominant AD (ADAD) is classified as a genetic alteration to the amyloid precursor protein (APP) or Presenilin (PSEN) genes [3]. The most notable characteristic of AD is the accumulation of amyloid beta plaques (Aβ) and neurofibrillary tangles (NFT) [4,5]. Normal amyloid peptides are not considered toxic and serve a physiological function, but the accumulation of misfolded amyloid proteins within tissues causes toxic and damaging effects on the organ [6]. The amyloid precursor protein (*APP*) gene, which is responsible for forming Aβ, is found on chromosome 21 [7]. APP translational products regulate cellular metabolism and mitochondrial function and are further cleaved into Aβ peptides [8,9,10]. The production of Aβ peptides increases under stress [11], which in mild increments can lead to enhanced long-term potentiation and hyperexcitability [12]. However, the overexpression of APPs leads to protein misfolding and toxic accumulation [6]. The triplication of the *APP* gene, and therefore overexpression of APP is thought to be one factor that drives the increased rate of AD in DS cases [13].

In addition to the extra copy of the *APP* gene leading to an increased penetrance of AD in DS, alterations in the mammalian target of rapamycin (mTOR) cellular signaling pathway may contribute to the increased systemic dysregulations and neurodegeneration seen in persons with DS. mTOR is known to play a key role in cellular signaling processes [14]; however, the exact mechanism by which mTOR dysregulation contributes to AD in DS is still unclear. Individuals with DS also have mitochondrial deficits that lead to oxidative stress along with increased inflammatory activators and decreased cell survival [15,16,17], which also play a key role in the mTOR pathway [14]. The two hallmark characteristics of AD, Aβ plaques and NFTs, are also strongly associated with mTOR activity [18,19,20]. The activation of mTOR promotes the accumulation of Aβs [21] and NFTs [22] while others suggest that the inactivation of mTOR may slow age-related conditions [23,24].

## 2. mTOR Pathway

**Figure 1:**
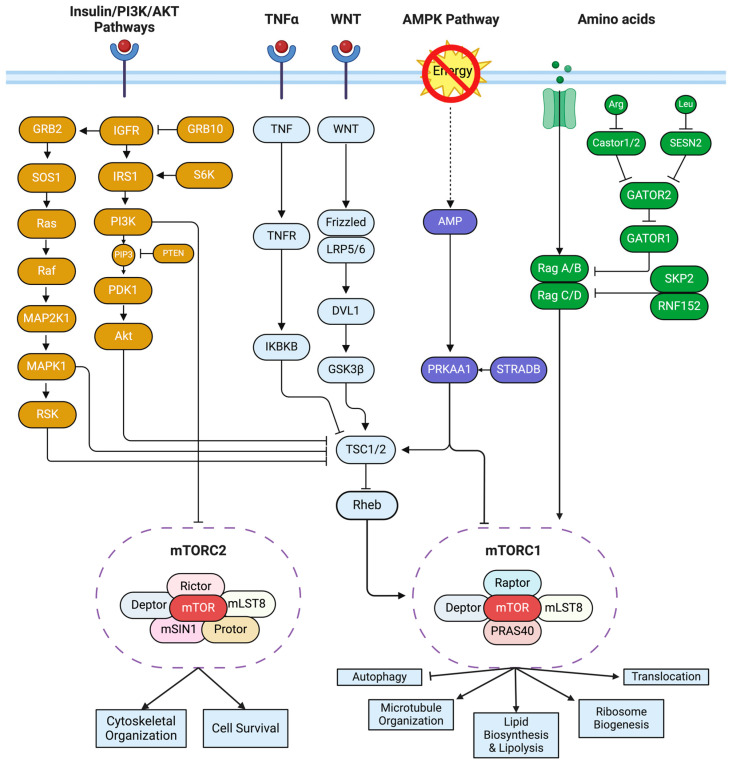
mTOR signaling pathway. The green pathway signifies the genes associated with the amino acid signaling pathway. The purple-colored genes are indicative of the AMPK pathway. The orange genes represent genes associated with the insulin/PI3K/AKT pathways. All genes in light blue are associated with the mTOR pathway, but not discussed within this review. The genes enclosed in dashed ovals represent the two mTOR complexes. This graph was created with BioRender.com and adapted from https://www.genome.jp/pathway/hsa04150 (accessed on 30 January 2024).

mTOR is a 289 kDa serine/threonine protein kinase that regulates and modulates protein synthesis, cytoskeletal organization, autophagy, and cell survival within eukaryotic cells [14]. mTOR, along with several proteins, form two different complexes, mTOR complex 1 (mTORC1), and mTOR complex 2 (mTORC2). mTORC1 comprises regulatory-associated protein of mTOR (rptor) and proline-rich Akt substrate 40 kDa (PRAS40/AKT1S1), see Figure 1. mTORC2 consists of rapamycin-insensitive companion of mTOR (rictor), mammalian stress-activated map kinase-interacting protein 1 (mSin1), and proline-rich protein 5 (Protor/PRR5). Both complexes contain the N-terminal DEP (Dishevelled, Egl-10, and Pleckstrin) domain-containing mTOR-interacting protein (Deptor), the mammalian homolog of protein Lethal with Sec Thirteen (mLST8), telomere length regulation protein (telo2), and TELO2-interacting protein 1 (TTI1). The upstream regulators of the mTOR pathway include growth factors, nutrients, and factors involved in hypoxic stress [25]. mTORC1 and mTORC2 act on downstream targets to modulate autophagy, microtubule organization, protein synthesis, and cell survival (Figure 1). However, the mechanisms by which mTORC2 works are less known than those mechanisms involving the mTORC1 complex [14]. A dysregulation of the mTOR pathways has been observed in age-related cognitive decline, as well as in individuals with AD and/or DS-AD [14,22,26,27]. Specifically, defects in autophagy in DS-AD may affect neuronal survival in the brain [28], but the activation of the mTOR pathway also has direct effects on the accumulation of amyloid and p-Tau [23,29,30]. Interestingly, the restoration of mTOR regulation with rapamycin restores several physiological deficits in DS mouse models [26,31,32] and increases longevity [33], strongly implicating this pathway for normal brain function. It is thought that the hyperactivation of the mTOR pathways leads to defects in autophagy. More studies are, however, needed to examine alterations in either of the two mTOR complexes which may lead to downstream or upstream dysfunction.

### 2.1. Insulin/mTOR Pathways

**Figure 2:**
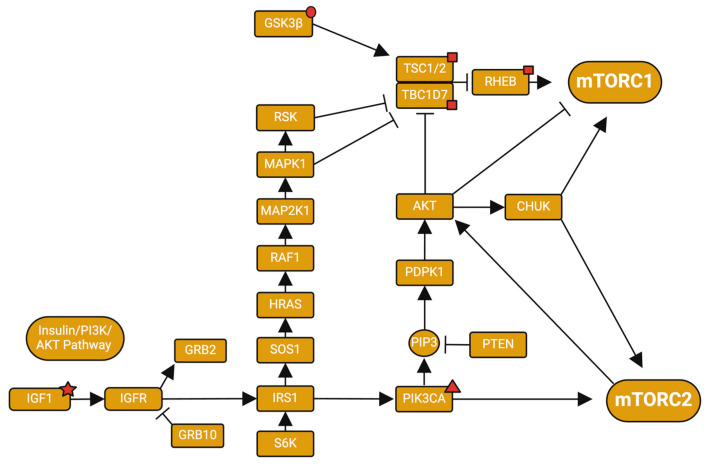
Insulin/PI3K/AKT signaling interactions with mTOR. Red shapes indicate genes/proteins discussed in the text. Created with BioRender.com. Adapted from https://www.genome.jp/pathway/hsa04150 (accessed on 30 January 2024).

Insulin/insulin-like growth factor 1 (IGF1) is an upstream modulator of both mTOR complexes (see Figure 2 red star). Insulin signaling influences mTORC1 signaling by modulating the PI3K/AKT axis, which in turn modulates the Tuberous sclerosis complex (TSC). TSC is a GTPase activator that modulates the Ras homolog enriched in brain (Rheb; see Figure 2 red square) GTPase, an activator of mTORC1 [34]. Similarly, the insulin/PI3K pathway allows PI3K to directly activate mTORC2 (see red triangle in Figure 2). The insulin/IGF/mTOR signaling pathway is a negative loop cycle that is essential for the growth, proliferation, and survival of cells [27]. mTORC1 is able to phosphorylate p70S6K, leading to the phosphorylation and subsequent degradation of insulin receptor substrate-1(IRS-1) [35]. The degradation of IRS-1 can lead to a downregulation of insulin activities [35] and insulin resistance [36]. A recent manuscript by Perluigi et al. [37] showed that exosomes derived from the blood of young children with DS revealed a significant increase in pIRS1^Ser636^, along with the hyperactivation of the Akt/mTOR/p70S6K axis downstream from IRS1, demonstrating an early dysregulation of the insulin/IGF/mTOR signaling pathway which could underlie some of the AD pathology observed with aging in DS in terms of the mTOR pathways and insulin resistance [38], and also at least partly intellectual disabilities observed in DS since IGF administration can rescue brain function in many neurodevelopmental disorders [39]. mTOR signaling also has the ability to be activated when insulin/IGF1 activity is inhibited [40,41]. Studies have associated lower levels of insulin and IGF1 with AD pathologies such as accelerated tau hyperphosphorylation [42,43,44], showing that an impairment of the insulin/IGF/mTOR signaling pathway can be linked to AD in the general population as well. These studies show the importance of homeostasis between the insulin and mTOR pathways and that slight disruptions can lead to detrimental effects. However, the importance of insulin and mTOR pathways in the context of DS-related alterations is not well understood.

### 2.2. PI3K/AKT/mTOR

The binding of growth factors to their respective receptors is followed by the activation of the phosphatidylinositol 3-kinase (PI3K)/Akt signaling [25,45], subsequently activating both mTORC1 and mTORC2. The activation of mTORC1 inhibits the function of ULK1 and Atg13, both regulators of autophagy [14]. The hyperactivation of this pathway and mTORC1 further suppresses autophagy genes/proteins leading to a decrease in autophagy. When autophagy is suppressed, debris within the brain or other tissue cannot be cleared sufficiently, leading to the accumulation and/or aggregation of misfolded proteins. A study by Perluigi et al. found hyperactivation of the PI3K/Akt/mTOR pathway in DS subjects with or without AD when compared to healthy controls [46]. As stated above, persons with DS carry three copies of the *APP* gene, leading to an overexpression of the APP protein and consequently also an increased production of Aβ peptides [7]. The dysfunction of autophagy via mTOR activation can lead to increased Aβ accumulation due to restricted clearance [21]. Aβ can in turn activate the PI3K/Akt signaling pathway, causing a further activation of mTORC1 and accumulation of plaques [47,48].

Akt is also a known regulator of GSK3β (see red circle in Figure 2), via phosphorylation of the inhibitory serine residue on GSK3β. GSK3β is an important kinase that regulates apoptosis, survival, memory formation, and neuronal development within the brain [49]. A study has shown higher levels of GSK3β expression in DS and DS-AD cases when compared to controls, as well as increased levels of GSK3β inhibitory phosphorylation [46]. GSK3β has the ability to phosphorylate Tau proteins and APP [50,51], so increased inhibition, like in DS-AD, should decrease Tau phosphorylation and APP aggregation, but that is not the case. The amount of GSK3β overexpression seen in these cases could outweigh the increased levels of GSK3β inhibition, ultimately increasing Tau and APP phosphorylation. Other factors, such as insulin, insulin-like growth factor, epidermal growth factor, mitogen-activated protein kinase, and BDNF also inhibit GSK3β activity [52,53,54]. GSK3β can inhibit phosphatase and tensin homolog (PTEN), activating Akt, which creates a negative feedback loop, inactivating GSK3β [55]. The heightened levels of GSK3β inhibitory phosphorylation seen in DS and DS-AD could be due to the aberrant Akt signaling suggested in other studies and should be investigated [56,57,58]. Other pathways associated with mTOR signaling should also be investigated due to the interconnected feedback seen within signaling pathways [49]. Dysregulation of the PI3K/AKT/mTOR signaling pathway and GSK3β could be one cause of decreased cell survival and hyperphosphorylation of tau and NFT deposits seen in DS and AD but this connection has not been examined closely to date.

### 2.3. AMPK Signaling

**Figure 3:**
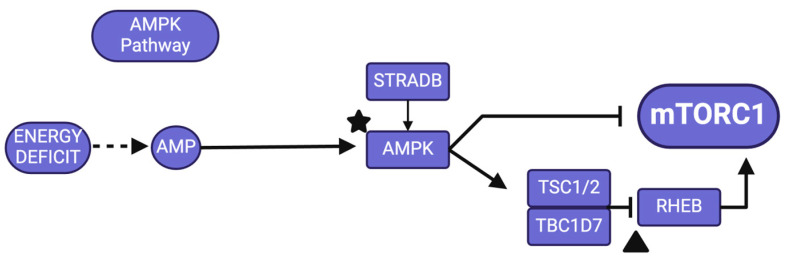
AMPK signaling pathway. Black shapes indicate genes/proteins discussed in text. Created with BioRender.com. Adapted from https://www.genome.jp/pathway/hsa04150 (accessed on 30 January 2024).

Adenosine monophosphate (AMP)-activated protein kinase (AMPK; see black star in Figure 3), an essential energy enzyme, manages cellular metabolism to sustain energy balance when intracellular adenosine triphosphate (ATP) levels decrease [27]. It is a tau kinase and can increase the phosphorylation of tau at Ser-262 and directly phosphorylate tau at Ser-396/404 and Thr-231 [14]. AMPK is also an upstream inhibitor of mTORC1 by directly inhibiting the complex, or by activating the TSC. TSC is formed by tuberous sclerosis 1 and 2 and TBC1 domain family member 7 (TBC1D7) and its activation directly inhibits Rheb, an activator of the mTORC1 complex (see black triangle in Figure 3). The activation of AMPK is essential in the initiation of autophagy through phosphorylation of Ulk1 whereas mTORC1 inhibits phosphorylation and weakens autophagy [59]. The induction of autophagy via AMPK activation may decrease levels of Aβ [60,61] and inhibition via mTORC1 activation may increase Aβ accumulation [19,21]. A study using the Ts65Dn DS mouse model demonstrated decreased levels of pAMPK/AMPK in the hippocampus [62], supporting the notion that AMPK failure plays a key role in DS-AD. However, this has not been demonstrated in the hippocampus of humans with DS and/or AD yet.

### 2.4. Amino Acid Regulation

Nutrients are important for regulating cellular processes. mTORC1 senses nutrients to modulate cell growth, autophagy, and other physiological processes [63]. While growth factors regulate mTOR, the signaling pathway cannot fully function without amino acid supplementation [64]. Meng and collaborators have shown that 10 of the 20 standard amino acids (Ala, Arg, Asn, Gln, His, Leu, Met, Ser, Thr, and Val) activate mTORC1 in both mouse and human tissue cultures via two distinct pathways [63]. These experiments were performed using mouse embryonic fibroblasts and human embryonic kidney 293A (HEK293A) cells in vitro. Based on their results, the same amino acids that regulate mTORC1 activity also promote the translocation of mTOR to lysosomal membranes. It is difficult to draw conclusions regarding differences between human and mouse-derived cells in terms of the mTORC1 pathway. Le Douce et al. demonstrated that the oral dietary supplementation of L-serine prevented both synaptic and behavioral deficits in 3xTg-AD mice, suggesting that this supplementation could be used for human AD therapy [65], and has not been tried in individuals with DS to our knowledge. Previous studies have shown the need for further investigation to determine the mechanisms by which amino acid intake influences cognitive health and to explore its potential as a therapeutic target for AD, since this would involve only dietary supplements that naturally occur in the human body [66].

**Figure 4:**
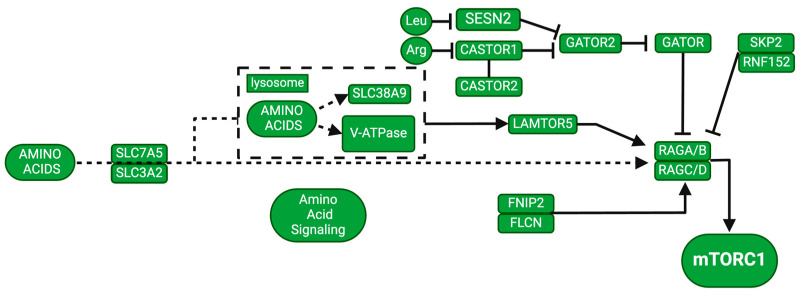
Amino acid pathway interaction with mTORC1. Created with BioRender.com. Adapted from https://www.genome.jp/pathway/hsa04150 (accessed on 30 January 2024).

Ras-related GTP-binding protein (Rag) is present in four forms (RagA-RagD) in mammals [67,68,69]. Amino acids act upon RAG to form its active conformation by loading RagA/B with GTP and RagC/D with GDP [70]. Active Rag can recruit inactive mTORC1 to the surface of lysosomes where Rheb then activates mTORC1 [64], meaning that amino acids can directly act on Rags to activate mTORC1, see Figure 4.

Rag activity is also regulated by GATOR (GAP Activity Towards Rags). GATOR is comprised of two subcomplexes: GATOR1 and GATOR 2 [71]. GATOR2 inhibits GATOR1 upon amino acid stimulation. GATOR2 activity is modulated by the binding of two different amino acids: leucine to Sestrin2 [72] and arginine binding to CASTOR1 [73] (Figure 4). Arginine binds and inhibits CASTOR1, activating GATOR2 and mTORC1 [73]. Conversely, GATOR2 is inhibited with amino acid depletion, and GATOR1 is activated.

Sestrin2 binds GATOR2 in the absence of leucine, resulting in the inhibition of GATOR2 and activation of GATOR1 [72]. Activated GATOR1 inactivates Rags via GTP hydrolysis, in turn inhibiting mTORC1 [71], meaning that deficient amounts of amino acids can inhibit mTORC1 activity. Studies have shown that autophagy is an important regulator of amino acid levels and defective cells cannot maintain physiological levels of amino acids to maintain homeostasis [74]. Aberrant mTORC1 activation leading to decreased autophagy within cells may decrease amino acid availability, creating a loop to further increase mTORC1 activation.

These basic physiological functions of mTOR have been studied since the early 1990s [75], and the mTOR complexes have been investigated since the early 2000s [76,77], but upstream and downstream signaling pathways have only recently been investigated in depth. The new methods described below can unearth even more important aspects of these signaling pathways and their role in neurodegenerative conditions such as DS-AD or AD.

## 3. Omics

“Omics” is a relatively new scientific approach that can provide substantial amounts of data at a particular level to represent the structure and function of a given biological system [78]. These relatively novel technologies can be used on a single cell or whole tissue level to map the physiological information of a given sample. Different methods of investigation include genomics (genetic material), transcriptomics (RNA transcripts), proteomics (expressed proteins), and metabolomics (metabolites) [78]. Some of these methods have been used to reveal signaling aberrations occurring in the brain of individuals with DS. These and additional, novel tools are discussed below.

### 3.1. Genomics

Genomics is the study of an organism’s complete set of genetic material (chromosomes, genes, and DNA) and how they interact with other genes and the environment. Genomics utilizes several techniques such as fluorescent in situ hybridization (FISH), karyotyping, and next-generation sequencing (NGS) techniques such as whole genome sequencing (WGS), targeted sequencing (TS), and whole exome sequencing (WES) to study the genome of an organism.

WGS is used to sequence the entirety of an organism’s genome, coding and non-coding regions, and provides a comprehensive approach for the discovery of novel mutations and variants that occur within the genome. TS is a more focused approach that can target specific genes or regions of a genome. This is a faster and more cost-effective method for the investigation of particular variations in genetic material. WES is limited to protein-coding regions of the genome but can be of use in identifying specific mutations in known protein pathways such as DS and mTOR. Genomics is a rapidly evolving field that will continue to provide insight into disease and drug development.

Genomics has been critical to the understanding, identification, and diagnosis of Down Syndrome (DS). The genomic signature of DS is a complete or partial replication of the DS critical region of chromosome 21 [79]. The triplication of chromosome 21 can be a complete trisomy, all cells in the body have three copies of chromosome 21, or mosaic, in which the individual has two differing cell lines in which some cells carry three copies of chromosome 21 and some carry the typical two copies. A translocation of the q-arm of chromosome 21 can also result in DS [80]. These alterations in chromosomes can be detected through NGS techniques, karyotyping, and FISH.

The sequencing of chromosome 21 provided several insights into DS pathology such as overexpression of interferon receptors (IFNAR1, IFNAR2, INFAG2, and Il-10B), increased production of amyloid peptides (APP and BACE2), and hyper-phosphorylation of Tau (DYRK1A and RCAN1) [81]. These expression profiles lead to AD pathology and systemic inflammation, two hallmark characteristics of DS [14].

Genomics has also given extensive information about the genes associated with AD, such as apolipoprotein E (APOE), BIN1, ABCA7, PICALM, MS4A4E/MS4A6A, CD2AP, CD33, EPHA1, CLU, CR1, and SORL1 all being associated with late-onset AD (LOAD) [82,83,84,85]. These genes are all linked to LOAD, however, not one causative gene has been identified [86]. Genomics for Autosomal Dominant AD has uncovered mutations within APP, presenilin 1 and 2 (PSEN1/2) genes, while sporadic AD (SAD) has been linked to polymorphism of the APOE gene [86]. With the APP gene residing on chromosome 21, most persons with DS will likely develop AD.

Yang et al. performed an extensive analysis of a cluster of genes associated with the mTOR pathway, including the gene responsible for producing the mTOR protein, MTOR. This study was performed in 48 different non-human mammals and identified 20 genes closely associated with the mTOR complexes. These genes were also associated with longevity in mammals. Of these, several amino-acid-related genes (SESN2, IRS1, and TTI1) showed mutations in favor of longevity [87]. Genes that are associated with enhanced autophagy, which also improves longevity, such as NRPL3 (GATOR 1), and Lamptor 2 and 4 (Lamptor/Ragulator) were also increased in mammals who had increased longevity and resistance to age-related diseases [87]. Performing this extensive analysis of the mTOR pathway on human DS, AD, and DS-AD brain tissue can lead to new insights into how the human genome mutates to produce these conditions. Identifying genetic markers within the mTOR pathway may lead to new or more in-depth diagnostic methods for these disorders.

### 3.2. Transcriptomics

One form of transcriptomics is single-cell RNA sequencing (scRNA-seq). scRNA-seq can be used to detect and analyze messenger RNA (mRNA) in biological samples at single-cell resolution to study cellular responses [88]. This is a critical methodology to analyze the cellular response of tissue to different disease states. A study examining DS fetal tissue and DS mouse models reported ribosome stoichiometry loss, mitochondrial disruptions, and cellular senescence throughout multiple cell types, specifically fibroblasts and neural progenitor cells in the DS tissue compared to age-matched controls [89]. Ribosome and protein production, mitochondrial function, and cellular proliferation are all maintained by the function of mTOR [90,91].

Another study by Qiu et al. also reported abnormal neural differentiation in patients with DS using scRNA-seq [92]. The findings reported inadequate neuronal formation in DS cases [92], which could be linked to the increase of glial fibrillary acid protein-positive cells, such as astrocytes, seen in 18–20 week fetal DS cases [93,94]. These studies show a link to aberrant fetal brain development in DS persons and a cellular differentiation imbalance from the beginning potentially leading to the brain abnormalities reported in children with DS [95,96,97,98].

Muza et al. reported in their study of hippocampal and prefrontal dynamics that Dp(10)2Yey mouse models showed upregulated signaling pathways involved with memory function, notably the mTOR signaling pathway when compared to age-matched wild-type littermates [99]. While scRNA-seq provides an abundance of data regarding the cellular function of a tissue sample, it cannot provide spatial information for that tissue, since spatial resolution is lost in the process.

Spatial transcriptomics was first introduced in 2016 [100] and involves a barcoded array surface to visualize and analyze the full genome, transcriptome, or proteome of mRNA in a tissue sample with full spatial resolution [100,101,102]. Spatial omics are continually advancing to explore the biological and physiological changes within diseased tissue. Miyoshi et al. spatially profiled human and mouse prefrontal cortical tissue sampled from multiple stages of clinical AD, AD in DS, 5xFAD mice, and wild-type mice. The study provides a collection of species-conserved amyloid-associated genes that can be used for further analysis into disease mechanisms at a cellular and spatial level [103]. In a study by Vermeulen et al., they investigated the genetic mutation of mTOR in focal cortical dysplasia type II. The results showed hyperactive mTOR pathways leading to the cortical disorganization of the diseased state [104]. Another study focusing on PI3K/mTOR signaling in pontine gliomas was able to map the signaling pathway utilizing spatial transcriptomics and analyzing disease dysfunction [105]. These initial studies demonstrate the powerful analyses that can be carried out using novel spatial transcriptomic methods, either in mouse models or the human brain. Importantly, spatial transcriptomics preserves the spatial resolution of tissues, allowing researchers to study gene expression patterns in specific neuronal populations and/or specific layers of cells, rather than just obtaining an average expression level across the entire brain region. In preliminary studies, we have examined expression profiles in discrete regions of the hippocampus in postmortem brain tissue from early- and late-onset AD versus DS-AD and identified cell-specific alterations in expression levels (Granholm oral communication, to be published elsewhere). However, to our knowledge, mTOR signaling has not been analyzed previously regarding DS or DS-AD using spatial transcriptomics. Spatially analyzing the disruption of mTOR within DS-AD cases could identify steps in the pathways that lead to new treatment and therapeutic methods to prevent the development of AD pathology in individuals with DS.

### 3.3. Proteomics

Proteomics is a technology in which mass spectrometry is used to profile the structure and function of proteins within a given sample. There are several types of proteomics, including functional, expressional, or structural, that can be used based on the data desired. However, due to the complexity of the protein profile, combining this method with another approach, such as transcriptomics or genomics, may provide a better understanding of the biological information provided [106]. A study conducted by Sullivan et al. assessed the circulating proteome in plasma from individuals with DS and found changes corresponding to chronic autoinflammation, specifically increased levels of Tumor necrosis factor-alpha (TNF-α) [107]. It has also been reported that TNF-α, an upstream modulator of mTOR, can influence the formation of Aβ plaques and NFTs [108,109,110]. Another study has mapped out the proteoforms of Aβ plaques with human AD samples [111]. Domenico et al. used proteomics to map HNE-modified proteins (these are proteins that have reacted with 4-hydroxynonenal (HNE), a toxic byproduct of lipid peroxidation) in DS mouse models after treatment with rapamycin, an mTOR antagonist [32]. The results showed a strong correlation between aberrant mTOR and HNE modifications within proteins. However, neither this study nor any other has specifically defined the proteasome of aberrant mTOR within humans with DS.

### 3.4. Metabolomics

Metabolomics is another powerful profiling technique that provides an in-depth profile of all metabolites and low-molecular-weight molecules present within a given specimen [112]. A multi-omics study reported distinct molecular profiles in AD cases associated with decreased cognitive function, early onset/death, and dysregulated neuronal/synaptic pathways [113]. A study conducted by Novotny et al. also identified 133 unique metabolites shared between autosomal dominant AD, carriers of risk variants in TREM2, and sporadic AD (sAD) as well as 16 metabolites that are altered throughout the progression of AD [114]. These metabolites include amino acids, such as asparagine, methionine, serine, threonine, and valine [114], which play a role in mTORC1 activation [63]. Many pathways involving the biosynthesis, metabolism, and degradation of different amino acids, such as leucine, histidine, alanine, etc., were also altered in ADAD when compared to control cases [114]. Patterson et al. also performed metabolomics in Ts65Dn mice after rapamycin treatment and found that treatment of young and aged mice lowered several DS-related metabolites, norepinephrine and homovanillic acid, in DS models to the levels found in the respective control models, leading to an increased lifespan. The researchers also noted changes to dopamine, kynurenine, and 13 other unidentified metabolites in response to rapamycin treatment in both young and aged mice [115]. Not surprisingly, the data provided by these studies indicate that metabolic changes occur with AD and DS-AD. These changes should be explored with in-depth analyses into pathway and regional dysfunctions associated with disease states and can now be performed in the human brain and be associated with spatial resolution to identify specific neuronal or glial populations affected by the disease in the human brain.

## 4. Conclusions

The relationship between DS and AD reveals significant insights into the pathological mechanisms underlying neurodegenerative diseases. The triplication of chromosome 21 in DS, leading to an increased production of Aβ plaques due to the overexpression of the APP gene, establishes a genetic basis for the elevated risk of AD in individuals with DS. Additionally, the dysregulation of the mTOR pathway plays a crucial role in increasing AD pathology in DS. Several other pathways, such as insulin, PI3K/AKT, AMPK, and amino acid signaling, are involved in the development of AD pathology in DS. All of these pathways can be examined using novel omics approaches that have not yet been undertaken for DS-AD pathology.

mTOR signaling influences various cellular processes including autophagy, protein synthesis, and cell survival. The hyperactivation of mTORC1, influenced by upstream regulators such as insulin/IGF1, PI3K/Akt, AMPK, and amino acids, suppresses autophagy, leading to the accumulation of neurotoxic Aβ plaques and NFTs.

New research utilizing omics technologies such as genomics, transcriptomics, proteomics, and metabolomics, provides a deeper understanding of the molecular and cellular alterations in DS and AD. The genomic analysis of DS, AD, and DS-AD can show genetic alterations that lead to the onset of these conditions and are a potential target for more in-depth diagnostic methods. Single-cell RNA sequencing and spatial transcriptomics reveal disruptions in ribosomal function, mitochondrial dynamics, and cellular senescence, highlighting the intricate network of dysregulated pathways in these diseases. Proteomics studies identify alterations in protein expression and post-translational modifications, implicating inflammatory processes and metabolic dysregulation in the progression of AD in DS. Metabolomic research has shown specific metabolites, including many amino acids (Ala, Arg, Asn, Gln, His, Leu, Met, Ser, Thr, and Val), dopamine, kynurenine, and other unidentified substances associated with AD pathology. Further investigating and understanding of how these metabolites lead to or are altered during diseased states could potentially lead to novel treatments for the disease mechanisms causing downstream effects, such as mTORC1 hyperactivation, autophagy dysregulation, or excess Aβ production seen in AD, DS, and DS-AD.

Future research should focus on pinpointing the precise mechanisms by which mTOR signaling contributes to the pathogenesis of DS and AD, with an emphasis on spatially resolved omics analyses. The next steps would be to investigate pathway dysregulations in subregions of tissue and how surrounding cell types are affected. Such studies could reveal novel therapeutic targets and strategies aimed at modulating mTOR activity and restoring cellular homeostasis. Ultimately, understanding the complex relationship between genetic, cellular, and molecular factors in DS and AD will pave the way for innovative approaches to the diagnosis, treatment, and prevention of neurodegenerative conditions.

## Data Availability

Data pertaining to this review are available via previously published work, which is indicated in the publication list.
